# The Role of Surface-Modified Titanium Scaffolds and Implants in Tissue Engineering for Bone Healing in Maxillofacial and Reconstructive Surgery: Short title: The Role of Surface-Modified Titanium Scaffolds and Implants in Maxillofacial and Reconstructive Surgery

**DOI:** 10.31661/gmj.v15i.4174

**Published:** 2026-08-16

**Authors:** Mohammed Abbas Jasim, Shaikhaliev Astemir Ikramovich

**Affiliations:** ^1^ Department of Maxillofacial Surgery, N.N.Bazhanov Institute of Dentistry, E.V.Borovsky FSAOU HE First MSMU , I.M. Sechenov of the Ministry of Health of Russia, Russia

**Keywords:** Titanium, Surface Modification, Osseointegration, Maxillofacial Reconstruction, Tissue Engineering, 3D PSrinting

## Abstract

Titanium and titanium alloys are widely used in maxillofacial and reconstructive surgery because they combine mechanical strength, corrosion resistance, manufacturability, and long-term biocompatibility. Their applications include dental implants, mandibular reconstruction plates, titanium mesh, patient-specific implants, orbital reconstruction systems, cranial implants, zygomatic implants, and additively manufactured porous scaffolds. Although titanium provides reliable mechanical stability, its biological performance depends strongly on the surface–tissue interface rather than on bulk material properties alone. Surface chemistry, oxide-layer characteristics, roughness, wettability, and micro/nanotopography influence protein adsorption, osteoblast adhesion, macrophage phenotype, angiogenesis, bacterial adhesion, and osseointegration. This narrative review critically synthesizes current knowledge on surface-modified titanium scaffolds and implants for bone healing in maxillofacial and reconstructive surgery, with emphasis on biological mechanisms, clinical relevance, translational limitations, and future directions. The available evidence supports the concept that optimized titanium surfaces can promote osteogenesis, vascularization, osteoimmunomodulation, and antibacterial activity, but clinical translation remains limited by heterogeneous surface characterization, simplified preclinical models, and insufficient long-term clinical data.

## Introduction

Titanium has become a foundational biomaterial in oral, maxillofacial, craniofacial, and reconstructive surgery because it provides stable fixation, favorable corrosion resistance, and acceptable biological tolerance in hard-tissue environments [1, 2, 3, 4]. The classical concept of osseointegration established that titanium can achieve direct structural and functional anchorage with living bone under appropriate biological and mechanical conditions [1, 2]. Subsequent research demonstrated that osseointegration is not governed only by implant shape or alloy composition but also by surface properties such as roughness, chemistry, wettability, oxide structure, and topography [3, 4]. This distinction is clinically important because maxillofacial implants are often placed in complex defects involving trauma, tumor resection, congenital deformity, infection, irradiation, grafting, or oral microbial exposure [5, 6, 7]. In maxillofacial reconstruction, titanium is used as an endosseous dental implant material, a load-bearing fixation material, a space-maintaining mesh, a patient-specific reconstructive implant, and a porous scaffold for bone ingrowth [7, 8, 9]. Conventional titanium can restore form and mechanical continuity, but biological integration requires favorable interactions among blood proteins, immune cells, osteogenic cells, endothelial cells, and microorganisms at the surface interface [10, 11, 12, 13, 14, 15, 16, 17]. Surface modification is therefore a central strategy for converting titanium from a relatively bioinert structural material into a biologically instructive interface for tissue engineering [18, 19, 20, 21, 22]. Surface-modified titanium should therefore be understood as a dynamic biointerface rather than a passive fixation material, because its surface chemistry, oxide structure, wettability, roughness, nanotopography, and porosity collectively regulate early protein adsorption, inflammatory signaling, osteogenic differentiation, vascularization, antibacterial defense, and long-term bone–implant contact [11, 12, 13, 14, 15, 16, 17, 18, 19, 20, 21, 22, 23, 24]. A conceptual framework illustrating these interconnected mechanisms is presented in Figure-1. This review discusses surface-modified titanium scaffolds and implants as multifunctional platforms for bone healing in maxillofacial and reconstructive surgery.

### Titanium in Maxillofacial and Reconstructive Surgery

Titanium dental implants remain the most established clinical example of load-bearing osseointegration in the craniofacial skeleton [3, 4]. Moderately rough and chemically activated dental implant surfaces generally show improved early bone response compared with historical machined surfaces, although surface roughness must be balanced against the risk of plaque accumulation when exposed to the oral cavity [3, 4]. Mandibular reconstruction plates restore continuity after trauma, tumor ablation, osteoradionecrosis, or congenital deformity correction, but their long-term success can be limited by infection, plate exposure, screw loosening, stress shielding, and fatigue fracture [7, 8]. Titanium mesh is used in guided bone regeneration, alveolar augmentation, orbital floor repair, cranial contour reconstruction, and defect containment because it provides space maintenance and structural support [6, 7]. Patient-specific titanium implants are increasingly used for maxillary, mandibular, zygomatic, orbital, and cranial reconstruction because digital workflows can improve anatomical fit, reduce intraoperative bending, and support more accurate restoration of complex skeletal contours [5, 6, 7]. Clinical studies of patient-specific titanium implants have reported encouraging outcomes in selected maxillofacial defects, but bone fusion, infection control, soft-tissue coverage, and long-term implant stability remain decisive clinical endpoints [6, 7]. In orbital reconstruction, patient-specific CAD/CAM titanium implants can improve positional accuracy compared with manually adapted or preformed implants in selected fracture patterns, although cost, availability, and workflow time remain practical limitations [25]. Three-dimensional printed porous titanium scaffolds extend this concept by combining patient-specific geometry with controlled porosity, pore interconnectivity, and surface architecture designed to support bone ingrowth [8, 9]. The main clinical challenge is that conventional titanium is mechanically reliable but not intrinsically osteoinductive [3, 10]. The biological response to titanium is shaped by the native oxide layer, adsorbed proteins, surface contamination, micro/nanoscale topography, wettability, and corrosion behavior [17, 18, 19, 20, 21]. These factors influence cell adhesion, inflammatory resolution, angiogenesis, bacterial colonization, and long-term bone–implant contact [11, 12, 13, 14]. Surface modification is therefore particularly relevant in maxillofacial surgery because oral and sinus microbial exposure, thin soft-tissue coverage, compromised vascularity, and functional loading can threaten implant integration [5, 15].

## Biological Basis of Titanium Surface Modification

**Table:** Table1. Key Mechanisms Influenced by Surface-Modified Titanium

Mechanism	Surface-dependent factors	Markers or outcomes	Surgical relevance
Protein adsorption	Chemistry, charge, wettability, roughness	Fibronectin, vitronectin, fibrinogen	Determines early cell-interface conditioning [10, 11]
Osteoblast adhesion	Micro/nanotopography, integrin ligand exposure	ALP, RUNX2, COL1, osteocalcin	Supports early bone formation [12, 13]
Osseointegration	Oxide layer, roughness, coating stability	BIC, removal torque, implant stability	Determines long-term fixation [1–4]
Angiogenesis	Porosity, VEGF delivery, ion release	VEGF, CD31, HIF-1α	Required for large defect healing [8, 9]
Osteoimmunomodulation	Hydrophilicity, nanotopography, ion release	M1/M2 markers, TNF-α, IL-10	Regulates inflammation and repair [14, 17]
Antibacterial effect	Anti-adhesive topography, Ag, Zn, Cu, chitosan	Biofilm biomass, bacterial viability	Crucial in oral and sinus exposure [15, 16]

**Table:** Table2. Major Titanium Surface Modification Strategies

Category	Examples	Main purpose	Key limitation
Mechanical	Sandblasting, machining, laser texturing	Roughness and interlocking	Plaque retention if exposed [3, 15]
Chemical	Acid etching, alkali treatment, silanization	Bioactivity and apatite formation	Reproducibility and aging [17, 18]
Electrochemical	Anodization, MAO, TiO₂ nanotubes	Oxide control, ion incorporation	Brittle coatings and release control [22]
Physical	Plasma spraying, PVD, UV treatment	Coating or activation	Delamination or aging [19]
Bioactive coatings	HA, CaP, collagen, chitosan, BMP-2, VEGF	Osteogenesis and angiogenesis	Dose, stability, regulation [24]
Nanostructures	Nanotubes, nanopores, nanoparticles	Cell signaling and drug loading	Parameter sensitivity [13, 22]
3D-printed scaffolds	Porous Ti6Al4V lattices	Patient-specific bone ingrowth	Fatigue and residual powder [8, 9]

**Figure-1:**
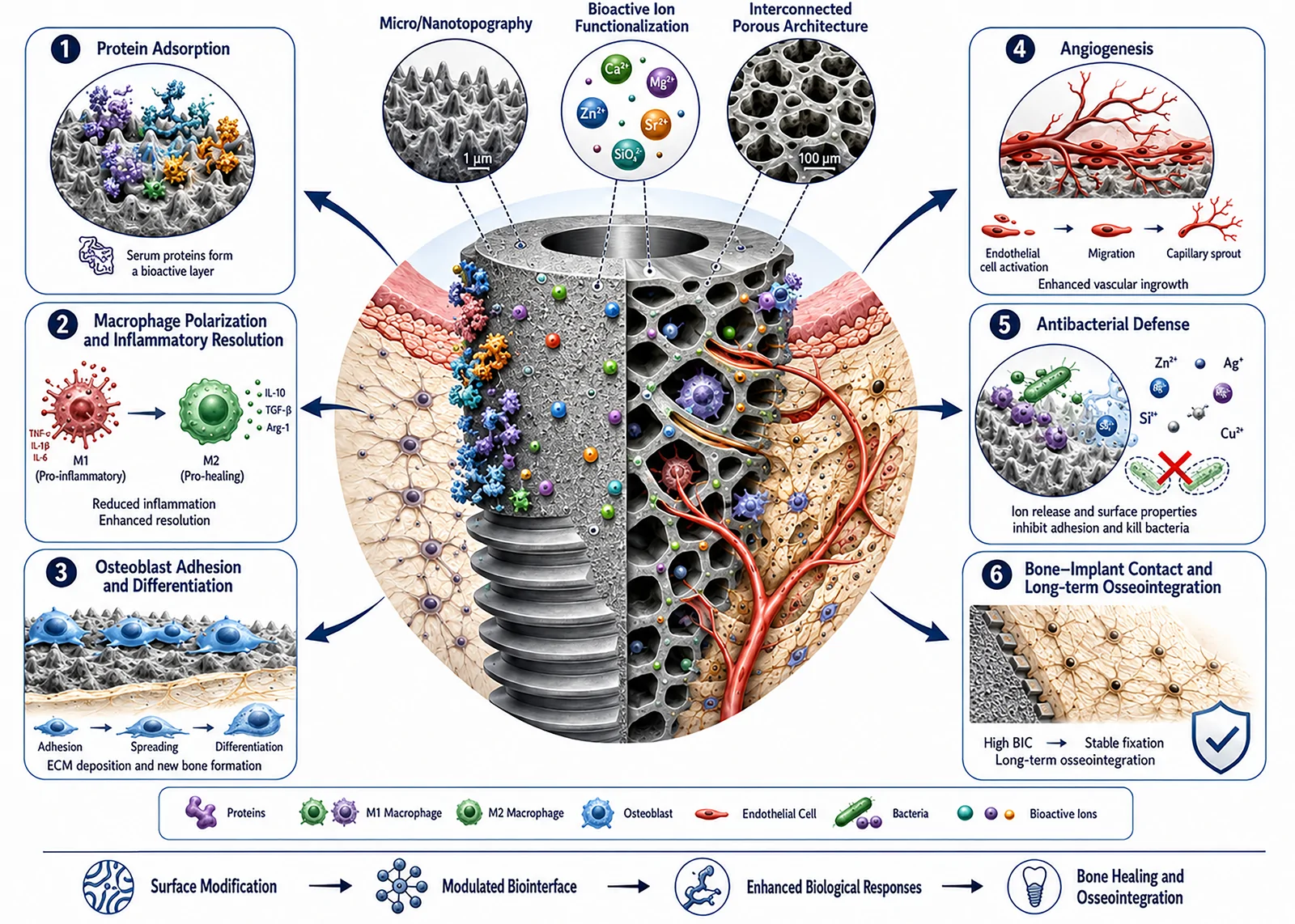
Figure 1. Effects of titanium surface modification on bone healing and osseointegration.

### Mechanical Surface Modification

Mechanical surface modification includes machining, polishing, sandblasting, laser texturing, and controlled micro/nano-roughening [17, 18, 19]. These methods primarily alter roughness, surface area, and mechanical interlocking between bone and titanium [3, 17]. Sandblasting and acid-etching combinations have been widely used to create moderately rough surfaces that enhance early bone response compared with machined surfaces [3, 4]. Laser texturing can generate more controlled microgrooves or hierarchical surface features than conventional subtractive methods, although scalability and cost must be considered [18, 19]. A major limitation of mechanical roughening is that surfaces favorable for osseointegration may also retain bacteria more readily when exposed to the oral environment [15, 16].

### Chemical Modification

Chemical modification includes acid etching, alkali treatment, hydrogen peroxide treatment, silanization, and chemical grafting [18]. Acid etching can remove contaminants and create micro-pitted topography that improves early bone response [3, 4]. Alkali treatment and hydrogen peroxide treatment can increase surface hydroxylation and promote apatite-forming ability under appropriate conditions [18, 19]. Silanization and chemical grafting allow attachment of peptides, polymers, growth factors, or antimicrobial molecules to titanium surfaces [23, 24]. The main limitation of chemical modification is the need for reproducible chemistry after sterilization, storage, surgical handling, and exposure to blood or saliva [19].

### Electrochemical Modification

Electrochemical modification includes anodization, micro-arc oxidation, plasma electrolytic oxidation, and TiO₂ nanotube formation [20, 21, 22]. Anodization can generate ordered TiO₂ nanotubes that provide nanoscale topographical cues and local reservoirs for antibiotics, growth factors, or anti-inflammatory agents [13]. TiO₂ nanotube arrays have been investigated because they can influence protein adsorption, osteoblast differentiation, antibacterial activity, and local therapeutic delivery [13, 22]. Micro-arc oxidation can create porous ceramic-like oxide layers and incorporate calcium, phosphate, magnesium, zinc, strontium, copper, or silver into the surface [20, 21]. Micro-arc oxidation is widely studied because it can improve bioactivity, corrosion resistance, roughness, and ion-mediated bone response, but coating brittleness, delamination, and ion-release control remain important limitations [21].

### Physical Modification

Physical modification includes plasma spraying, physical vapor deposition, ion implantation, laser ablation, and ultraviolet activation [18, 19]. Plasma spraying can deposit hydroxyapatite or calcium phosphate coatings that increase osteoconductivity [19]. Physical vapor deposition can generate thin functional layers such as titanium nitride, diamond-like carbon, or antibacterial metallic coatings [19]. Ion implantation can introduce biologically active or antibacterial elements into the near-surface region without forming a thick coating [17]. Ultraviolet activation can reduce hydrocarbon contamination and increase surface hydrophilicity, although biological effects may diminish with surface aging [19].

### Bioactive Coatings

Bioactive coatings include hydroxyapatite, calcium phosphate, collagen, chitosan, BMP-2, VEGF, RGD peptides, bioactive glass, and ion-doped inorganic layers [24]. Hydroxyapatite and calcium phosphate coatings support osteoconduction by mimicking the mineral phase of bone [17, 18, 19, 20, 21]. Collagen and RGD peptides support cell adhesion by presenting extracellular matrix-like biochemical cues [19]. BMP-2 and VEGF coatings aim to stimulate osteogenesis and angiogenesis, but clinical translation is complicated by dose control, burst release, cost, and regulatory classification [20, 21, 22]. Chitosan–hydroxyapatite coatings are frequently discussed because hydroxyapatite supports osteoconduction while chitosan can contribute biocompatibility, film-forming capacity, and antimicrobial activity [23, 24]. The main translational concerns for bioactive coatings are adhesion strength, sterilization stability, degradation behavior, cytotoxicity, mechanical durability, and reproducible manufacturing [17, 18, 19, 20].

### Nanostructured Titanium Surfaces

Nanostructured titanium surfaces include TiO₂ nanotubes, nanopores, nanoroughness, nanowires, nanoparticles, and hierarchical micro/nano surfaces [17, 22]. Cells sense nanoscale features through filopodia, integrins, membrane curvature, focal adhesions, and cytoskeletal tension [13, 17]. Nanostructures can modulate protein adsorption, osteoblast differentiation, macrophage phenotype, bacterial adhesion, and local drug release [13, 14, 15, 16, 17]. Nano-engineered titanium surfaces are especially relevant to craniofacial implants because they may support bone formation, soft-tissue integration, antibacterial defense, and localized therapeutic delivery [13]. The major limitation is that biological effects are highly sensitive to nanotube diameter, nanotube length, crystallinity, cleaning protocol, sterilization method, and storage conditions [17, 22].

3D-Printed Porous Titanium Scaffolds Additive manufacturing enables patient-specific titanium scaffolds with controlled porosity, pore size, strut thickness, lattice geometry, fixation points, and defect-specific morphology [6, 7, 8, 9]. Porous titanium scaffolds can reduce elastic modulus, improve mechanical compatibility with bone, and create interconnected pathways for bone and vessel ingrowth [8, 9]. Three-dimensional printed titanium grid scaffolds have shown potential for mandibular segmental defects because they can combine sufficient mechanical strength with pores that facilitate osteogenesis [8]. Microporous titanium alloy implants have also been reviewed as promising platforms for large segmental defects because pore architecture can influence elastic modulus, fatigue behavior, osteoconduction, angiogenesis, and mechanobiological signaling [9]. Post-printing surface treatment is usually necessary because additively manufactured titanium may contain residual powder, partially melted particles, surface contamination, and roughness features that are not biologically optimal [8, 9]. The future value of 3D-printed porous titanium in maxillofacial reconstruction will depend on integrating anatomical fit, mechanical safety, fatigue resistance, vascularized bone ingrowth, antibacterial function, and reproducible post-processing [17]. Because each surface-modification strategy offers distinct advantages and limitations, selection of an appropriate approach should be guided by the anatomical site, mechanical demand, infection risk, soft-tissue condition, and intended regenerative function [17, 18, 19, 20, 21, 22, 23, 24]. The principal surface-modification categories and their translational implications are summarized in Table2.

### Current Challenges and Knowledge Gaps

Surface characterization remains inconsistent across titanium studies, and many reports do not fully describe roughness, chemistry, wettability, oxide composition, coating thickness, crystallinity, sterilization effects, or aging behavior [17, 18]. This lack of standardization limits reproducibility and makes it difficult to compare biological outcomes across surface-modification strategies [20, 21]. Many in vitro studies rely on osteoblast monocultures, although maxillofacial healing involves immune cells, endothelial cells, fibroblasts, saliva, oral microorganisms, mechanical loading, and sometimes irradiated or scarred tissue [14, 15, 16]. Antibacterial studies frequently use short-term single-species assays, whereas clinical maxillofacial implants are exposed to multispecies oral biofilms and repeated mechanical cleaning [15, 16]. Clinical evidence for patient-specific and 3D-printed titanium implants is promising but still dominated by retrospective studies, small cohorts, and heterogeneous outcome reporting [9, 25]. Mechanical and biological goals may conflict because increasing porosity can improve tissue ingrowth but reduce fatigue strength, and increasing roughness can improve osseointegration but increase bacterial retention [8, 9, 15]. Ion-doped and antibacterial coatings require careful optimization because excessive silver, copper, zinc, or other ion release can impair osteoblast viability or soft-tissue compatibility [21].

### Future Perspectives

Future titanium implants for maxillofacial reconstruction should be designed as multifunctional biointerfaces rather than as passive fixation devices [18, 19, 20, 21, 22, 23, 24]. Hierarchical micro/nano surfaces may combine mechanical interlocking with nanoscale control of protein adsorption, cell adhesion, macrophage response, and antibacterial behavior [16, 17]. Immunomodulatory surfaces should aim to guide the temporal sequence of healing by permitting early antimicrobial inflammation and then promoting macrophage transition toward regenerative phenotypes [14, 17]. Porous titanium scaffolds should be engineered to support vascular invasion because large craniofacial defects require oxygen delivery, nutrient supply, and osteoprogenitor recruitment throughout the scaffold volume [9, 17]. Antibacterial designs should be tested against clinically relevant multispecies oral biofilms rather than only against planktonic single-strain cultures [15, 16]. Digital design, finite element analysis, topology optimization, and additive manufacturing can help balance patient-specific geometry, mechanical loading, pore architecture, and biological integration [8, 9]. Clinical translation will require standardized fatigue testing, corrosion testing, surface characterization, sterilization validation, biofilm testing, animal models relevant to craniofacial biology, and long-term human follow-up [21].

## Conclusion

Surface-modified titanium scaffolds and implants are central to modern maxillofacial and reconstructive tissue engineering because they combine structural stability with the potential to direct biological healing. Their biological performance depends on the surface-mediated regulation of protein adsorption, osteoblast differentiation, osseointegration, angiogenesis, immune response, and bacterial adhesion. Mechanical, chemical, electrochemical, physical, bioactive-coating, nanostructuring, and additive-manufacturing approaches each offer distinct advantages and limitations.

Micro-arc oxidation, TiO₂ nanotubes, chitosan–hydroxyapatite coatings, ion-doped surfaces, and 3D-printed porous titanium scaffolds are particularly relevant to craniofacial bone healing because they can integrate osteogenic, angiogenic, immunomodulatory, and antibacterial functions. Despite strong mechanistic and preclinical support, the field requires more clinically realistic models, standardized surface characterization, and long-term comparative studies before many advanced surfaces can be adopted routinely.

The next generation of titanium implants should be developed as patient-specific, mechanically safe, biologically active, and infection-resistant platforms for complex craniofacial reconstruction.

## Conflict of Interest

The author declares that there is no conflict of interest related to this manuscript.

## AI Disclosure Statement

During the preparation of this manuscript, AI–based language assistance (ChatGPT) was used to support manuscript restructuring, language polishing, grammar correction, and improvement of clarity and flow. The authors reviewed, edited, and approved all AI-assisted content and take full responsibility for the accuracy, integrity, and final version of the manuscript.
